# Supplementary material for: Freshwater reservoir offsets on radiocarbon-dated dog bone from the headwaters of the St. Lawrence River, USA
Source: PeerJ. 2019 Jun 25;7:e7174. doi: 10.7717/peerj.7174 (PMC6598671; doi:10.7717/peerj.7174)
Supplement: Table S1 [file peerj-07-7174-s001.docx]

John P. Hart, Robert S. Feranec, Timothy J. Abel, Jessica Vavrasek

Freshwater Reservoir Offsets on Radiocarbon-Dated Dog Bone from the St. Lawrence River Headwaters, USA

**Supplemental Table S1. MixSIAR model results.**

**7 Source Bayesian (MixSIAR) model for individual Dogs**

***Canis lupus familiaris* (204721-A41925-118)**

**Mean SD 2.5% 5% 25% 50% 75% 95% 97.5%**

**Bear-Deer** 0.093 0.076 0.003 0.006 0.034 0.074 0.136 0.251 0.283

**Maize** 0.432 0.065 0.275 0.323 0.401 0.441 0.474 0.520 0.536

**HighNfish** 0.070 0.060 0.002 0.005 0.024 0.054 0.100 0.192 0.223

**LowNfish** 0.112 0.101 0.003 0.007 0.037 0.083 0.157 0.312 0.370

**MidNfish** 0.090 0.077 0.004 0.007 0.031 0.069 0.128 0.248 0.286

**Smherbivore** 0.103 0.082 0.003 0.006 0.038 0.086 0.152 0.264 0.304

**Turkey** 0.100 0.084 0.003 0.006 0.033 0.078 0.144 0.272 0.311

***Canis lupus familiaris* (204717-A73725-3)**

**Mean SD 2.5% 5% 25% 50% 75% 95% 97.5%**

**Bear-Deer** 0.092 0.081 0.003 0.005 0.030 0.070 0.130 0.253 0.297

**Maize** 0.272 0.076 0.092 0.129 0.226 0.282 0.325 0.379 0.397

**HighNfish** 0.157 0.107 0.009 0.016 0.072 0.141 0.226 0.350 0.401

**LowNfish** 0.136 0.117 0.003 0.008 0.044 0.105 0.197 0.368 0.429

**MidNfish** 0.151 0.122 0.005 0.009 0.054 0.122 0.220 0.383 0.448

**Smherbivore** 0.083 0.073 0.002 0.005 0.028 0.064 0.119 0.220 0.267

**Turkey** 0.109 0.095 0.004 0.008 0.037 0.083 0.154 0.298 0.359

***Canis lupus familiaris* (204715-A73725-1)**

**Mean SD 2.5% 5% 25% 50% 75% 95% 97.5%**

**Bear-Deer** 0.070 0.060 0.002 0.004 0.024 0.055 0.103 0.185 0.214

**Maize** 0.434 0.071 0.263 0.307 0.400 0.446 0.480 0.530 0.544

**HighNfish** 0.120 0.088 0.004 0.009 0.047 0.103 0.175 0.283 0.319

**LowNfish** 0.109 0.100 0.003 0.006 0.035 0.080 0.156 0.305 0.368

**MidNfish** 0.121 0.104 0.004 0.008 0.038 0.094 0.178 0.335 0.380

**Smherbivore** 0.063 0.058 0.002 0.004 0.020 0.046 0.089 0.175 0.211

**Turkey** 0.083 0.072 0.003 0.006 0.029 0.063 0.117 0.227 0.268

***Canis lupus familiaris* (199807-9103-1)**

**Mean SD 2.5% 5% 25% 50% 75% 95% 97.5%**

**Bear-Deer** 0.123 0.098 0.004 0.008 0.046 0.103 0.176 0.322 0.367

**Maize** 0.299 0.067 0.148 0.178 0.261 0.306 0.346 0.396 0.411

**HighNfish** 0.073 0.064 0.002 0.004 0.026 0.056 0.104 0.203 0.234

**LowNfish** 0.126 0.111 0.004 0.008 0.043 0.094 0.181 0.358 0.409

**MidNfish** 0.099 0.087 0.004 0.006 0.033 0.075 0.144 0.276 0.323

**Smherbivore** 0.153 0.110 0.006 0.013 0.065 0.134 0.218 0.359 0.404

**Turkey** 0.126 0.107 0.004 0.008 0.043 0.096 0.185 0.345 0.396

***Canis lupus familiaris* (199804-70908-1)**

**Mean SD 2.5% 5% 25% 50% 75% 95% 97.5%**

**Bear-Deer** 0.052 0.046 0.001 0.003 0.016 0.039 0.075 0.145 0.172

**Maize** 0.609 0.060 0.478 0.510 0.579 0.615 0.648 0.691 0.703

**HighNfish** 0.076 0.060 0.003 0.006 0.030 0.064 0.109 0.197 0.223

**LowNfish** 0.074 0.070 0.002 0.004 0.023 0.054 0.101 0.209 0.260

**MidNfish** 0.078 0.068 0.002 0.005 0.027 0.059 0.112 0.205 0.241

**Smherbivore** 0.052 0.047 0.002 0.003 0.016 0.039 0.074 0.142 0.174

**Turkey** 0.059 0.054 0.002 0.003 0.018 0.043 0.085 0.168 0.203

***Canis lupus familiaris* (199803-28082-1)**

**Mean SD 2.5% 5% 25% 50% 75% 95% 97.5%**

**Bear-Deer** 0.084 0.071 0.003 0.006 0.028 0.064 0.125 0.221 0.256

**Maize** 0.458 0.065 0.308 0.340 0.427 0.465 0.500 0.548 0.561

**HighNfish** 0.079 0.065 0.003 0.006 0.030 0.062 0.114 0.206 0.244

**LowNfish** 0.104 0.093 0.004 0.007 0.034 0.079 0.150 0.283 0.338

**MidNfish** 0.096 0.083 0.004 0.006 0.033 0.073 0.139 0.264 0.305

**Smherbivore** 0.090 0.073 0.004 0.007 0.033 0.073 0.129 0.232 0.275

**Turkey** 0.089 0.080 0.002 0.005 0.026 0.067 0.129 0.242 0.285

***Canis lupus familiaris* (199801-9091-2)**

**Mean SD 2.5% 5% 25% 50% 75% 95% 97.5%**

**Bear-Deer** 0.091 0.075 0.003 0.006 0.032 0.072 0.133 0.239 0.275

**Maize** 0.375 0.070 0.207 0.248 0.338 0.384 0.422 0.470 0.484

**HighNfish** 0.106 0.082 0.004 0.009 0.042 0.089 0.149 0.268 0.309

**LowNfish** 0.116 0.103 0.004 0.008 0.038 0.088 0.166 0.324 0.375

**MidNfish** 0.120 0.099 0.004 0.009 0.044 0.095 0.175 0.315 0.362

**Smherbivore** 0.089 0.074 0.003 0.006 0.033 0.071 0.130 0.231 0.268

**Turkey** 0.103 0.088 0.003 0.006 0.035 0.079 0.149 0.272 0.319

***Canis lupus familiaris* (199800-42163)**

**Mean SD 2.5% 5% 25% 50% 75% 95% 97.5%**

**Bear-Deer** 0.067 0.055 0.002 0.004 0.023 0.053 0.098 0.174 0.203

**Maize** 0.542 0.061 0.409 0.440 0.512 0.549 0.582 0.625 0.637

**HighNfish** 0.083 0.066 0.003 0.005 0.031 0.068 0.121 0.215 0.245

**LowNfish** 0.087 0.082 0.003 0.006 0.029 0.063 0.121 0.255 0.305

**MidNfish** 0.090 0.076 0.003 0.006 0.031 0.072 0.129 0.245 0.285

**Smherbivore** 0.060 0.054 0.002 0.004 0.020 0.045 0.087 0.168 0.200

**Turkey** 0.071 0.063 0.002 0.004 0.023 0.054 0.102 0.193 0.232

***Canis lupus familiaris* (199799-41436)**

**Mean SD 2.5% 5% 25% 50% 75% 95% 97.5%**

**Bear-Deer** 0.075 0.062 0.003 0.005 0.025 0.057 0.109 0.202 0.231

**Maize** 0.493 0.064 0.345 0.384 0.462 0.501 0.535 0.578 0.596

**HighNfish** 0.082 0.068 0.003 0.006 0.029 0.066 0.117 0.216 0.255

**LowNfish** 0.097 0.089 0.003 0.006 0.031 0.072 0.134 0.274 0.329

**MidNfish** 0.093 0.080 0.003 0.006 0.032 0.072 0.133 0.253 0.299

**Smherbivore** 0.076 0.065 0.002 0.005 0.026 0.060 0.107 0.204 0.241

**Turkey** 0.084 0.072 0.003 0.006 0.028 0.066 0.122 0.227 0.265

***Canis lupus familiaris* (199798-9114)**

**Mean SD 2.5% 5% 25% 50% 75% 95% 97.5%**

**Bear-Deer** 0.120 0.096 0.004 0.008 0.043 0.099 0.174 0.308 0.353

**Maize** 0.326 0.067 0.166 0.205 0.289 0.334 0.372 0.419 0.433

**HighNfish** 0.072 0.062 0.002 0.005 0.024 0.056 0.102 0.194 0.229

**LowNfish** 0.122 0.109 0.004 0.007 0.040 0.091 0.175 0.350 0.417

**MidNfish** 0.098 0.086 0.003 0.006 0.033 0.076 0.139 0.270 0.324

**Smherbivore** 0.143 0.107 0.005 0.010 0.056 0.124 0.211 0.347 0.392

**Turkey** 0.119 0.100 0.004 0.007 0.041 0.093 0.169 0.317 0.363

**3 Source Bayesian (MixSIAR) models for individual Dogs**

***Canis lupus familiaris* (204721-A41925-118)**

**Mean SD 2.5% 5% 25% 50% 75% 95% 97.5%**

**Maize** 0.469 0.064 0.322 0.356 0.435 0.475 0.512 0.562 0.576

**Deer** 0.322 0.136 0.034 0.065 0.232 0.342 0.422 0.516 0.544

**Fish** 0.209 0.154 0.008 0.017 0.086 0.178 0.303 0.506 0.554

***Canis lupus familiaris* (204717-A73725-3)**

**Mean SD 2.5% 5% 25% 50% 75% 95% 97.5%**

**Maize** 0.312 0.084 0.112 0.154 0.267 0.323 0.370 0.433 0.451

**Deer** 0.282 0.159 0.020 0.036 0.160 0.275 0.396 0.553 0.602

**Fish** 0.405 0.189 0.057 0.092 0.271 0.397 0.538 0.730 0.790

***Canis lupus familiaris* (204715-A73725-1)**

**Mean SD 2.5% 5% 25% 50% 75% 95% 97.5%**

**Maize** 0.47 0.072 0.304 0.339 0.429 0.478 0.520 0.569 0.583

**Deer** 0.21 0.126 0.013 0.025 0.107 0.198 0.301 0.427 0.468

**Fish** 0.32 0.154 0.032 0.062 0.208 0.326 0.430 0.564 0.615

***Canis lupus familiaris* (199807-9103-1)**

**Mean SD 2.5% 5% 25% 50% 75% 95% 97.5%**

**Maize** 0.339 0.073 0.173 0.214 0.297 0.347 0.389 0.446 0.464

**Deer** 0.433 0.164 0.049 0.097 0.337 0.464 0.548 0.657 0.691

**Fish** 0.228 0.182 0.009 0.019 0.086 0.177 0.324 0.603 0.669

***Canis lupus familiaris* (199804-70908-1)**

**Mean SD 2.5% 5% 25% 50% 75% 95% 97.5%**

**Maize** 0.636 0.059 0.502 0.530 0.604 0.642 0.675 0.721 0.734

**Deer** 0.156 0.094 0.008 0.015 0.077 0.154 0.227 0.313 0.336

**Fish** 0.208 0.117 0.012 0.028 0.117 0.205 0.292 0.403 0.446

***Canis lupus familiaris* (199803-28082-1)**

**Mean SD 2.5% 5% 25% 50% 75% 95% 97.5%**

**Maize** 0.493 0.063 0.349 0.384 0.456 0.499 0.536 0.585 0.599

**Deer** 0.276 0.132 0.024 0.046 0.174 0.289 0.380 0.474 0.503

**Fish** 0.232 0.152 0.011 0.023 0.106 0.216 0.340 0.505 0.550

***Canis lupus familiaris* (199801-9091-2)**

**Mean SD 2.5% 5% 25% 50% 75% 95% 97.5%**

**Maize** 0.413 0.072 0.243 0.283 0.374 0.421 0.463 0.514 0.531

**Deer** 0.287 0.147 0.025 0.045 0.175 0.293 0.399 0.519 0.555

**Fish** 0.300 0.170 0.025 0.043 0.169 0.289 0.421 0.592 0.651

***Canis lupus familiaris* (199800-42163)**

**Mean SD 2.5% 5% 25% 50% 75% 95% 97.5%**

**Maize** 0.573 0.06 0.437 0.465 0.538 0.577 0.615 0.662 0.678

**Deer** 0.192 0.11 0.011 0.022 0.104 0.187 0.276 0.373 0.401

**Fish** 0.235 0.13 0.017 0.032 0.130 0.233 0.329 0.452 0.489

***Canis lupus familiaris* (199799-41436)**

**Mean SD 2.5% 5% 25% 50% 75% 95% 97.5%**

**Maize** 0.527 0.063 0.387 0.424 0.494 0.533 0.569 0.613 0.629

**Deer** 0.234 0.121 0.017 0.031 0.139 0.239 0.328 0.420 0.449

**Fish** 0.238 0.143 0.018 0.031 0.122 0.224 0.339 0.485 0.529

***Canis lupus familiaris* (199798-9114)**

**Mean SD 2.5% 5% 25% 50% 75% 95% 97.5%**

**Maize** 0.364 0.071 0.204 0.237 0.326 0.370 0.412 0.468 0.483

**Deer** 0.407 0.158 0.048 0.099 0.310 0.434 0.521 0.623 0.657

**Fish** 0.228 0.177 0.011 0.020 0.087 0.186 0.329 0.579 0.645
