# Supplementary material for: Freshwater reservoir offsets on radiocarbon-dated dog bone from the headwaters of the St. Lawrence River, USA
Source: PeerJ. 2019 Jun 25;7:e7174. doi: 10.7717/peerj.7174 (PMC6598671; doi:10.7717/peerj.7174)
Supplement: Supplemental Information 2 [file peerj-07-7174-s003.docx]

John P. Hart, Robert S. Feranec, Timothy J. Abel, & Jessica Vavrasek

Freshwater Reservoir Offsets on Radiocarbon-Dated Dog Bone from the St. Lawrence River Headwaters, USA

**Supplemental Code S1. OxCal QCL Run Files.**

**QCL run file for a single uniform phase model with using dates on maize and deer bone collagen.**

Plot()

{

Sequence()

{

Boundary("Start Northern New York");

Phase("Northern New York")

{

R_Date("UGAMS-34185",314,22);

R_Date("UGAMS-31486",336,22);

R_Date("UGAMS-30327",357,23);

R_Date("UGAMS-30326",313,23);

R_Date("UGAMS-34187",337,22);

R_Date("UGAMS-34188",319,22);

R_Date("UGAMS-30328",309,23);

R_Date("UGAMS-30931",310,20);

R_Date("UGAMS-34445",320,20);

R_Date("UGAMS-34446",315,20);

R_Date("UCIAMS-199806",410,15);

R_Date("UGAMS-30325",349,23);

R_Date("UGAMS-30324",344,23);

R_Date("UGAMS-30323",382,23);

R_Date("UGAMS-30322",351,23);

R_Date("UGAMS-26745",392,22);

R_Date("UGAMS-26744",359,22);

R_Date("Beta-148524",400,50);

R_Date("UGAMS-26743",399,22);

R_Date("UGAMS-26742",383,22);

R_Date("UCIAMS-199802",410,15);

R_Date("UCIAMS-199805",405,15);

R_Date("UCIAMS-205977",345,20);

R_Date("UCIAMS-205978",345,15);

R_Date("UCIAMS-205979",365,15);

R_Date("UCIAMS-205969",370,15);

R_Date("UCIAMS-205970",365,15);

R_Date("UCIAMS-205971",380,15);

R_Date("UCIAMS-205972",335,20);

R_Date("UCIAMS-205973",355,20);

R_Date("UCIAMS-205974",410,20);

R_Date("UCIAMS-205975",360,15);

R_Date("UCIAMS-204714",485,25);

R_Date("UCIAMS-204718",420,25);

R_Date("UCIAMS-204719",410,25);

R_Date("UCIAMS-204720",445,25);

R_Date("UCIAMS-204722",415,30);

R_Date("UCIAMS-207138",415,15);

R_Date("UGAMS-37380",353,20);

R_Date("UGAMS-37381",392,20);

R_Date("UGAMS-37382",363,20);

R_Date("UGAMS-37383",315,20);

R_Date("UGAMS-37384",500,25);

Date("Date Northern New York");

};

Boundary("End Northern New York");

Before("Glass Beads",C_Date(1600,10));

};

};

**QCL run file for a single uniform phase model with dates on maize and deer and dog bone collagen.**

Plot()

{

Sequence()

{

Boundary("Start Northern New York");

Phase("Northern New York")

{

R_Date("UGAMS-34185",314,22);

R_Date("UGAMS-31486",336,22);

R_Date("UGAMS-30327",357,23);

R_Date("UGAMS-30326",313,23);

R_Date("UGAMS-34187",337,22);

R_Date("UGAMS-34188",319,22);

R_Date("UGAMS-30328",309,23);

R_Date("UGAMS-30931",310,20);

R_Date("UGAMS-34445",320,20);

R_Date("UGAMS-34446",315,20);

R_Date("UCIAMS-199806",410,15);

R_Date("UGAMS-30325",349,23);

R_Date("UGAMS-30324",344,23);

R_Date("UGAMS-30323",382,23);

R_Date("UGAMS-30322",351,23);

R_Date("UGAMS-26745",392,22);

R_Date("UGAMS-26744",359,22);

R_Date("Beta-148524",400,50);

R_Date("UGAMS-26743",399,22);

R_Date("UGAMS-26742",383,22);

R_Date("UCIAMS-199802",410,15);

R_Date("UCIAMS-199805",405,15);

R_Date("UCIAMS-205977",345,20);

R_Date("UCIAMS-205978",345,15);

R_Date("UCIAMS-205979",365,15);

R_Date("UCIAMS-205969",370,15);

R_Date("UCIAMS-205970",365,15);

R_Date("UCIAMS-205971",380,15);

R_Date("UCIAMS-205972",335,20);

R_Date("UCIAMS-205973",355,20);

R_Date("UCIAMS-205974",410,20);

R_Date("UCIAMS-205975",360,15);

R_Date("UCIAMS-204714",485,25);

R_Date("UCIAMS-204718",420,25);

R_Date("UCIAMS-204719",410,25);

R_Date("UCIAMS-204720",445,25);

R_Date("UCIAMS-204722",415,30);

R_Date("UCIAMS-207138",415,15);

R_Date("UGAMS-37380",353,20);

R_Date("UGAMS-37381",392,20);

R_Date("UGAMS-37382",363,20);

R_Date("UGAMS-37383",315,20);

R_Date("UGAMS-37384",500,25);

R_Date("UCIAMS-199814",460,15);

R_Date("UCIAMS-199798",535,20);

R_Date("UCIAMS-199801",580,15);

R_Date("UCIAMS-199803",510,20);

R_Date("UCIAMS-204721",550,25);

R_Date("UCIAMS-199800",490,15);

R_Date("UCIAMS-199807",515,15);

R_Date("UCIAMS-204715",610,25);

R_Date("UCIAMS-204717",705,30);

R_Date("UCIAMS-199799",525,15);

Date("Date Northern New York");

};

Boundary("End Northern New York");

Before("Glass Beads",C_Date(1600,10));

};

};
